# Integrated Volatilomics, Metabolomics, and Transcriptomics Implicate PLD–PA–LPAR Signaling in Rabbit Meat Flavor Divergence

**DOI:** 10.3390/ani16203162

**Published:** 2026-10-09

**Authors:** Xiaojing Liu, Ce Liu, Gongyan Liu, Shuxia Gao, Liya Bai, Zhenyan Yang, Dalong Zhang, Haitao Sun

**Affiliations:** 1 Key Laboratory of Livestock and Poultry Multi-omics of MARA, Institute of Animal Science and Veterinary Medicine, Shandong Academy of Agricultural Sciences, Jinan 250100, China; lemo0613@126.com (X.L.); liuceshiyan@163.com (C.L.); gongyanliu@foxmail.com (G.L.); shuxiagao_2004@163.com (S.G.); bailiya_2005@163.com (L.B.); 2 Jinan Academy of Agricultural Sciences, Jinan 250136, China; lwyangzhenyan@163.com (Z.Y.); jnzhangdalong@163.com (D.Z.)

**Keywords:** rabbit meat flavor, volatilomics, metabolomics, transcriptomics, O2PLS, hexanal

## Abstract

Chinese indigenous rabbit breeds are highly prized for their unique meat flavor, yet the molecular mechanisms underlying breed-specific flavor profiles remain incompletely understood. This study characterized the longissimus dorsi muscle of two indigenous Chinese black rabbit breeds, Laiwu Black (LW) and Minxinan Black (MX), through integrated analysis of volatilomics (HS-SPME-GC-MS), nontargeted metabolomics (LC-MS/MS), and transcriptomics (RNA-seq). LW rabbits displayed significantly increased intramuscular fatty acid content, characterized by elevated levels of de novo lipogenesis markers and polyunsaturated fatty acids (PUFAs), including linoleic acid (C18:2n6). In contrast, MX rabbits exhibited higher concentrations of elongation products such as C22:5n3 and C22:5n6. ROAV analysis identified hexanal, the primary oxidation product of linoleic acid (C18:2n6), as the most discriminative key odorant between breeds. Transcriptomic profiling revealed 2747 differentially expressed genes, with KEGG pathway analysis identifying PPAR signaling, phospholipase D signaling, and cAMP signaling as significantly enriched pathways. This study employed multi-omics integration through O2PLS analysis, Pearson correlation, and KEGG co-enrichment to identify a central molecular discriminator: the PLD–PA–LPAR lipid signaling axis. Key regulatory genes such as *PLA2G7*, *PLD2*, and LPAR family members were found to connect glycerophospholipid metabolism with breed-specific fatty acid partitioning. These findings identify a gene–lipid–volatile network in which PPAR signaling-mediated lipogenesis is associated with polyunsaturated fatty acid (PUFA) substrates for hexanal generation, while PLD–PA–LPAR signaling may influence the allocation of fatty acyl-CoA between phospholipid turnover and triacylglycerol storage. Together, these mechanisms are associated with the breed-specific aroma profiles in rabbit meat.

## Simple Summary

Chinese indigenous rabbit breeds are renowned for their delicious meat, yet the molecular mechanisms underlying breed-specific flavor differences remain poorly understood. In this study, we compared the muscle tissues of two Chinese black rabbit breeds—Laiwu Black and Minxinan Black—using three omics approaches: volatilomics (to analyze aroma compounds), metabolomics (to identify small molecules), and transcriptomics (to assess gene activity). Our findings revealed that Laiwu Black rabbits accumulated higher levels of intramuscular fat, particularly linoleic acid (a polyunsaturated fatty acid), while Minxinan Black rabbits exhibited greater production of longer-chain fatty acids. Hexanal, a volatile compound formed during the oxidation of linoleic acid, emerged as the key aroma contributor distinguishing the two breeds. By integrating these multi-omics datasets, we identified a gene network linked to fat metabolism and cellular signaling. This network appears to regulate whether fats are utilized for membrane synthesis or energy storage, thereby shaping the distinct flavor profiles of each breed’s meat. These results illuminate the interplay between genes, lipids, and volatile compounds in defining breed-specific characteristics, offering insights that could guide breeding strategies to enhance meat quality and deliver more flavorful, high-quality rabbit products.

## 1. Introduction

Rabbit meat is valued for its favorable nutritional profile, notably characterized by high protein content (20–22%), low fat levels (5–8%), and lower cholesterol compared to other commonly consumed meats such as beef, pork, and chicken [1]. In addition to its nutritional value, the sensory attributes of meat, particularly its aroma, are a key factor influencing consumer preference and market acceptance across diverse livestock species [2,3]. Despite their culinary significance, the molecular mechanisms underlying flavor formation in rabbit meat, particularly in Chinese indigenous breeds, remain poorly understood. Chinese indigenous rabbit breeds, such as Laiwu Black (LW) and Minxinan Black (MX), are renowned for their distinct flavor profiles and high-quality meat characteristics. However, the molecular pathways underlying breed-specific aroma and taste traits remain largely elusive, hindering the development of targeted genetic and nutritional interventions to enhance rabbit meat quality.

Meat aroma is predominantly formed during thermal processing through complex chemical reactions, with lipid oxidation and the Maillard reaction serving as the primary pathways [4]. Unsaturated fatty acids, particularly linoleic acid (C18:2n6) and α-linolenic acid (C18:3n3), serve as key precursors that undergo lipid peroxidation during cooking, generating a wide range of volatile organic compounds (VOCs), including aldehydes, alcohols, ketones, and esters [5]. Hexanal, a primary oxidation product of linoleic acid, is a key contributor to the characteristic meaty and grassy aromas in cooked meat [6]. Consequently, the fatty acid composition of intramuscular lipids, particularly the levels and proportions of polyunsaturated fatty acids (PUFAs), is considered a key determinant of the volatile flavor profile. This, in turn, significantly influences the perceived meat quality [5].

The application of omics technologies has significantly advanced our understanding of the key molecular determinants underlying meat quality. Transcriptomic profiling has facilitated the identification of genes and pathways linked to intramuscular fat deposition, fatty acid metabolism, and muscle fiber type differentiation [7,8]. Metabolomics provides a complementary perspective by directly analyzing the metabolic end products of gene expression and environmental influences, including flavor-related metabolites and their precursors. Recent studies have shown the value of integrated multi-omics approaches in unraveling gene–metabolite networks that regulate key rabbit meat quality traits. Kuang et al. [9] used transcriptomic, proteomic, and metabolomic analyses to identify molecular regulatory networks linked to tryptophan metabolism and extracellular matrix (ECM)-related pathways as key differentiating factors between Shuxing No. 1 and IRA rabbit breeds. Liu et al. [10] demonstrated through targeted metabolomics and gas chromatography–ion mobility spectrometry (GC–IMS) that the interaction between cooking method and breed significantly modulates the volatile flavor profiles of Chuanbai Rex and Hylan rabbits. However, studies integrating parallel volatilomics, metabolomics, and transcriptomics profiling with a formal multi-omics framework such as O2PLS to dissect the genetic and metabolic basis of breed-specific meat aroma differences remain limited in rabbit meat research.

In this study, two Chinese indigenous rabbit breeds with markedly distinct genetic backgrounds and divergent meat quality traits, Laiwu Black (LW) and Minxinan Black (MX), were selected. An integrated multi-omics approach was employed, combining GC-MS-based fatty acid profiling, HS-SPME-GC-MS-based volatilomics with ROAV-guided key odorant identification, LC-MS/MS-based untargeted metabolomics, and RNA-seq-derived transcriptomics on longissimus dorsi (LD) muscle samples. Multi-omics integration was achieved through three complementary analytical frameworks: O2PLS modeling, pairwise Pearson correlation network analysis, and KEGG co-enrichment analysis. The study aimed to elucidate the core molecular pathways and gene–metabolite interaction networks underlying breed-specific flavor compound accumulation in rabbit LD muscle.

## 2. Materials and Methods

### 2.1. Animals and Sample Preparation

A total of 20 healthy male rabbits at market weight (approximately 12 weeks of age) from two breeds were used in this study: Laiwu Black rabbits (LW, *n* = 10) and Minxinan Black rabbits (MX, *n* = 10). All rabbits were raised under identical husbandry conditions at Laiwu Black Rabbit Breeding Technology Co., Ltd. (Jinan, China) and received the same commercial diet ad libitum. Prior to slaughter, rabbits were fasted for 12 h with water deprivation. Animals were euthanized by intravenous anesthesia. Immediately after slaughter, longissimus dorsi (LD) muscle samples were dissected from the lumbar region between the 1st and 7th lumbar vertebrae. For fatty acid and volatile compound analyses, fresh LD samples were immediately processed. For RNA and metabolite extraction, additional LD samples were snap-frozen in liquid nitrogen and stored at −80 °C. Fatty acid profiling, volatile compound analysis, and transcriptomic sequencing were performed on all 20 samples (*n* = 10 per breed). For metabolomic profiling, one LW sample (LW10) was identified as a statistical outlier and excluded, resulting in final sample sizes of *n* = 9 (LW) and *n* = 10 (MX) for metabolomic analysis. The exclusion was based on a quantitative, per-breed criterion (Figure S1) rather than visual inspection of PLS-DA score plots. Specifically, on the log2-transformed, pareto-scaled metabolomics feature matrix (8450 features; all 20 samples fully observed), the Mahalanobis distance of each sample to its own breed-group centroid was computed in the top-5 principal-component space using leave-one-out (LOO) estimation of the covariance (so that an outlier cannot mask itself at *n* = 10) and compared with the 99th percentile of the χ^2^ distribution with 5 degrees of freedom (15.09). LW10 yielded D^2^ = 3277 more than 200-fold above the 99% threshold and ~30-fold larger than the next most distant LW sample (LW8, D^2^ = 108.7) unambiguously identifying it as an extreme outlier; the largest within-group D^2^ among all 10 MX samples was only 29.2 (two orders of magnitude smaller), so no MX sample met the exclusion criterion. Two further LW samples (LW2, D^2^ = 53.0; LW8, D^2^ = 108.7) nominally exceeded the 99% χ^2^ line but remained below 10-fold the threshold and below 1/30 of the LW10 distance and were therefore retained; LW10 was the sole extreme outlier by a wide margin. For the integrated O2PLS model (Section 2.6), the two omics blocks were kept sample-matched: LW10 was likewise excluded from the transcriptomic block, so the joint analysis used the matched 9 LW individuals for both blocks (the 10 MX samples were retained in both). The standalone transcriptomic differential analysis (DESeq2) retained all 10 LW samples, because no outlier was detected in the transcriptomic PCA/QC; the reduction to *n* = 9 applies only to the integration step requiring sample-matched blocks. All animal procedures were approved by the Institutional Animal Care and Use Committee of Shandong Academy of Agricultural Sciences (Approval No. IASVM-2024-040) and conducted in accordance with the relevant guidelines and regulations.

### 2.2. Analysis of Free Fatty Acids Content

Free fatty acids were extracted using a modified isopropanol/n-hexane (2:3, *v*/*v*) protocol with an internal standard (0.2 mg/L). Each sample (approximately 1 g of homogenized LD muscle) underwent two extraction cycles involving vortex-assisted homogenization, mechanical disruption, and ultrasonic treatment at 4 °C. The pooled organic fractions were concentrated under a gentle nitrogen stream and derivatized with methanol/trimethylsilyl diazomethane (1:2, *v*/*v*) for 30 min at ambient temperature. The resulting fatty acid methyl esters (FAMEs) were dried under nitrogen, reconstituted in n-hexane, clarified via centrifugation (12,000× *g*, 5 min), and analyzed by GC-MS.

Chromatographic separation was performed on an Agilent 7890B gas chromatograph coupled with a 5977B mass selective detector (Agilent Technologies, Santa Clara, CA, USA), equipped with a DB-FastFAME capillary column (60 m × 0.25 mm × 0.25 μm; Agilent J&W) (Agilent Technologies, Santa Clara, CA, USA). Samples were injected in split mode at a ratio of 5:1, using helium as the carrier gas at a constant flow rate of 1 mL/min. The column temperature program was as follows: initial temperature of 50 °C (held for 1 min), ramped to 175 °C at 25 °C/min, then to 200 °C at 4 °C/min, and finally to 230 °C at 3 °C/min (held for 5 min). The injector and transfer line temperatures were maintained at 240 °C; the ion source and quadrupole temperatures were set at 150 °C and 230 °C, respectively. Electron impact ionization was conducted at 70 eV, with mass spectra acquired in full-scan (*m*/*z* 33–400) and selected ion monitoring (SIM) modes. Fatty acids were identified by comparing retention times and mass spectra with authentic FAME standards (Supelco 37-Component FAME Mix; Sigma Aldrich, St. Louis, MO, USA) and quantified by internal standard calibration.

### 2.3. Analysis of Volatile Flavor Compounds

For headspace volatile analysis, approximately 0.8 g (±16 mg) of homogenized LD muscle was transferred to a 20 mL headspace vial. An aliquot of 10 μL 2-octanol (10 mg/mL in deionized water) was added as an internal standard. A separate vial containing an n-alkane mixture (C7–C30, 10 mg/L each) was prepared for linear retention index (LRI) calibration. Volatile compounds were extracted using a PAL RSI 85 autosampler (CTC Analytics, Zwingen, Switzerland) equipped with a 50/30 μm divinylbenzene/carboxen/polydimethylsiloxane (DVB/CAR/PDMS) SPME fiber (Supelco, Bellefonte, PA, USA). The extraction protocol included pre-equilibration at 60 °C for 15 min with agitation, headspace adsorption for 30 min at 60 °C, and thermal desorption in the GC injector at 250 °C for 4 min in splitless mode. It should be noted that the LD muscle was not cooked prior to volatile analysis; samples were analyzed fresh/frozen, and the 60 °C treatment refers solely to HS-SPME headspace pre-equilibration and adsorption during extraction, not to cooking. Volatile compounds were therefore captured from the raw muscle matrix.

Volatile analysis was performed on an Agilent 7890B GC coupled to a 5977B MSD (Agilent Technologies), fitted with a DB-WAX capillary column (60 m × 0.25 mm × 0.25 μm; Agilent J&W). Helium was used as the carrier gas at 1 mL/min (constant flow), with a purge flow of 3 mL/min. The oven temperature program was 40 °C (held for 4 min), increased to 245 °C at 5 °C/min, and held at 245 °C for 5 min. The injector and transfer line were maintained at 250 °C; the ion source at 150 °C and quadrupole at 230 °C. Electron impact ionization was applied at 70 eV. Data were acquired in full-scan mode (*m*/*z* 20–400) after a 2.37 min solvent delay.

Raw data were processed using LECO ChromaTOF software (version 4.3X; LECO Corporation, St. Joseph, MI, USA) for baseline correction, deconvolution, peak alignment, and library matching. Volatile compounds were provisionally identified by comparing mass spectra with the NIST 14 [11] mass spectral library (National Institute of Standards and Technology, Gaithersburg, MD, USA) and confirmed by matching LRI values with published LRI databases. Semiquantification was performed using the internal standard method, with the relative content of each compound expressed as its peak area ratio relative to 2-octanol.

For key odorant identification, the relative odor activity value (ROAV) was calculated as previously described [12]. Briefly, the odor activity value (OAV) of each volatile compound was calculated as the ratio of its mean relative content (normalized to internal standard) to its reported odor threshold in water. The compound exhibiting the highest OAV was assigned a ROAV of 100, and the ROAVs of all other compounds were proportionally computed. Compounds with ROAV ≥ 1 were considered to contribute significantly to the overall aroma profile of rabbit meat.

### 2.4. Untargeted Metabolomics Profiling

For metabolomic profiling, 50 mg of frozen LD muscle was homogenized in 500 μL ice-cold 80% (*v*/*v*) methanol and incubated at −20 °C for 30 min to precipitate proteins. After centrifugation at 20,000× *g* for 15 min at 4 °C, the supernatant was transferred to a fresh tube and dried under reduced pressure (CentriVap, Labconco, Kansas City, MO, USA). The dried extract was reconstituted in 100 μL 80% methanol, vortexed, and centrifuged at 12,000× *g* for 5 min. A quality control (QC) sample was prepared by pooling 10 μL from each sample extract and was injected periodically (every 10 analytical samples) to monitor system stability.

LC-MS/MS analysis was performed on a Thermo Q-Exactive Orbitrap mass spectrometer (Thermo Fisher Scientific, Waltham, MA, USA) coupled to a Vanquish UHPLC system. Chromatographic separation was achieved on an ACQUITY UPLC HSS T3 column (2.1 × 100 mm, 1.8 μm; Waters Corporation, Milford, MA, USA) maintained at 40 °C. Mobile phase A consisted of 0.1% formic acid in water, and mobile phase B consisted of 0.1% formic acid in acetonitrile. The gradient program was 0–2 min, 2% B; 2–11 min, 2–50% B; 11–15 min, 50–98% B; 15–18 min, 98% B; 18–18.1 min, 98–2% B; 18.1–22 min, 2% B, at a flow rate of 0.3 mL/min. Mass spectra were acquired in both positive and negative electrospray ionization (ESI) modes over an *m*/*z* range of 80–1200 at a resolution of 70,000 for full MS and 17,500 for MS/MS. The top 10 most intense ions in each full scan were subjected to higher-energy collisional dissociation (HCD) fragmentation.

Raw LC-MS data were processed using the XCMS package (version 3.18.0) in R (version 4.3.0) for peak detection, retention time correction, and feature grouping and alignment [13]. Features with missing values exceeding 20% across samples and those with a QC relative standard deviation (RSD) exceeding 30% were excluded. Metabolite annotation was performed by matching accurate mass (mass error ≤ 10 ppm) and MS/MS fragmentation spectra against the Human Metabolome Database (HMDB; https://hmdb.ca, accessed on 11 July 2025) and the Kyoto Encyclopedia of Genes and Genomes (KEGG; https://www.kegg.jp, accessed on 11 July 2025) compound databases, supplemented with an in-house spectral library.

### 2.5. RNA Extraction, Sequencing, and Transcriptome Data Analysis

Total RNA was extracted from approximately 50 mg of frozen LD muscle using TRIzol reagent (Invitrogen, Carlsbad, CA, USA) following the manufacturer’s protocol. RNA purity and concentration were assessed using a NanoDrop 2000 spectrophotometer (Thermo Fisher Scientific), and RNA integrity was evaluated on an Agilent 2100 Bioanalyzer (Agilent Technologies) with the RNA 6000 Nano Kit. All samples had an RNA integrity number (RIN) ≥ 7.0.

cDNA libraries were constructed using the TruSeq Stranded mRNA Library Prep Kit (Illumina, San Diego, CA, USA) following the manufacturer’s protocol. Library quality and concentration were verified on the Agilent 2100 Bioanalyzer. Paired-end (2 × 150 bp) sequencing was performed on an Illumina NovaSeq 6000 platform by LC-Bio Technologies (Hangzhou, China).

Raw sequencing reads were quality-filtered using fastp (version 0.23.2) to remove adapter sequences, low-quality reads (Phred score < 20), and reads with ambiguous bases. Clean reads were aligned to the rabbit reference genome (OryCun2.0; GCA_000003625.1) using HISAT2 (version 2.2.1) with default parameters [14]. Gene-level read counts were quantified using featureCounts (Subread package, version 2.0.3) against the OryCun2.0 gene annotation (Ensembl release 106). Genes with a sum of read counts < 10 across all samples were excluded. Differential expression analysis between LW and MX breeds was performed using DESeq2 (version 1.42.0) [15] with the threshold set at |log_2_ fold change| > 1 and Benjamini–Hochberg adjusted *p* value (FDR) < 0.05. The OryCun2.0 assembly (GCA_000003625.1) with the Ensembl release 106 gene annotation was used because it is the most widely used rabbit reference genome and provides the most complete and best-curated gene annotation among the rabbit assemblies available at the time of the analysis, which is a prerequisite for reliable read alignment and gene-level differential expression analysis.

All raw and processed RNA-seq data have been deposited in the Genome Sequence Archive (GSA) at the National Genomics Data Center (https://ngdc.cncb.ac.cn/gsa) (accessed on 11 July 2025) under accession number CRA027674 [16].

### 2.6. RT-qPCR Validation

RT-qPCR validation was performed on the same frozen LD muscle samples used for transcriptome sequencing (*n* = 10 per breed). Total RNA (extracted with TRIzol reagent and quality-assessed on a NanoDrop 2000, as described above) was reverse-transcribed into cDNA using a PrimeScript RT Reagent Kit (Takara, Dalian, China) following the manufacturer’s instructions. RT-qPCR was performed on a Bio-Rad CFX96 real-time PCR system (Bio-Rad Laboratories, Hercules, CA, USA) using SYBR Green Master Mix (Takara), with GAPDH as the reference gene. Each sample was run in technical triplicate. Primer sequences for the six target genes (*PLA2G7*, *PLD2*, *LPAR1*, *LPAR4*, *ELOVL5*, and *DGAT2*) and *GAPDH* are listed in Supplementary Table S7. Relative expression levels were calculated using the 2^−ΔΔCt^ method. Differences in relative expression between breeds were assessed by two-tailed Welch’s *t*-test (*n* = 10 per breed), and *p* < 0.05 was considered statistically significant.

### 2.7. Multi-Omics Integration

Multi-omics integration was conducted through three complementary analytical modules:

Two-way orthogonal partial least squares (O2PLS) modeling. An O2PLS model was constructed using the OmicsPLS package (version 2.1.0) in R [17,18], with KEGG annotated DEGs as the X-block and KEGG-mapped differentially abundant metabolites (DAMs; VIP > 1 and *p* < 0.05 from OPLS-DA) as the Y-block. The optimal number of joint components, X-orthogonal components, and Y-orthogonal components were determined by 7-fold cross-validation. The joint component loadings were ranked to identify the top contributing genes (X-loadings) and metabolites (Y-loadings) driving inter-breed variation in the joint latent space. Variance explained (R^2^X, R^2^Y, R^2^Ycor) was reported.

The statistical significance of the O2PLS joint components was further assessed by a permutation test (200 iterations; Figure S2). For each iteration, the sample pairing between the transcriptomic and metabolomic blocks was randomly permuted while the within-block structure of each omic was preserved, the O2PLS model (two joint components; 0 X-orthogonal and 2 Y-orthogonal components, as selected by 7-fold cross-validation) was refitted, and the test statistic—the sum of squared correlations between the two joint score vectors (S)—was recorded. The observed statistic (S = 1.77) exceeded all 200 permuted values (null mean = 1.21), giving a permutation *p* = 0.005 and confirming that the joint components anchoring the PLD–PA–LPAR axis are significant beyond chance. In the refitted joint model, the correlated fractions explained R^2^Xcor = 0.30 and R^2^Ycor = 0.42 of the variance in the transcriptomic and metabolomic blocks, respectively (Y-orthogonal R^2^ = 0.16). The test was executed locally in Python 3.13.14 (NumPy 2.5.3, SciPy 1.18.1) using a NIPALS-based O2PLS implementation with PCA compression to 15 components per block and log2 + pareto scaling, on the 9 LW + 10 MX sample-matched individuals used for the joint model (LW10 excluded from both blocks).

Pairwise Pearson correlation network. Pairwise Pearson correlations were calculated between all DEGs and DAMs using the cor.test function in R (version 4.3.1). Gene–metabolite pairs with |r| > 0.8 and *p* < 0.01 were selected for network construction. Network topology analysis identified hub genes and hub metabolites based on their total number of significant associations (degree). The correlation network was visualized using Cytoscape (version 3.10.0).

KEGG co-enrichment analysis. DEG-based KEGG pathway enrichment and DAM-based KEGG pathway enrichment were performed independently using the cluster Profiler package (version 4.10.0) in R [19] with the rabbit gene set as the background. Pathways reaching *p* < 0.05 in both DEG and DAM-based analyses were designated as co-enriched pathways, indicating convergence of transcriptomic and metabolomic signals at the pathway level.

### 2.8. Statistical Analysis

All statistical analyses were performed in R (version 4.3.0; R Foundation for Statistical Computing, Vienna, Austria). Data are presented as mean ± standard deviation (SD). Comparisons between two groups were performed using Student’s *t*-test or Welch’s *t*-test when the assumption of equal variance was violated, as assessed by Levene’s test. The normality of residuals was assessed by the Shapiro–Wilk test. Statistical significance was defined as *p* < 0.05, with *p* < 0.01 annotated where applicable. Principal component analysis (PCA), partial least squares-discriminant analysis (PLS-DA), and orthogonal partial least squares-discriminant analysis (OPLS-DA) for metabolomic data were performed using the ropls package (version 1.34.0) in R. Model validation was performed by 200-iteration permutation testing. Graphical outputs were generated using ggplot2 (version 3.5.0) and GraphPad Prism (version 8.0; GraphPad Software, San Diego, CA, USA). The multiple-testing status of the exploratory comparisons was made explicit: the 26 individual fatty-acid *t*-tests reported in Table 1 were not corrected for false discovery rate (FDR) and should be interpreted as exploratory, and the 281,622 pairwise gene–metabolite correlations in the Pearson network were filtered using nominal thresholds (*p* < 0.05/0.01/0.001) rather than FDR-adjusted values. Given the modest per-breed sample size (*n* = 9–10), false positives at nominal thresholds cannot be excluded; the central conclusions of this study therefore rest on the convergence of three independent methods (O2PLS, Pearson network, and KEGG co-enrichment) rather than on any single nominal *p* value.

## 3. Results

### 3.1. The Composition of Fatty Acids in the Two Breeds

**Figure 1:**
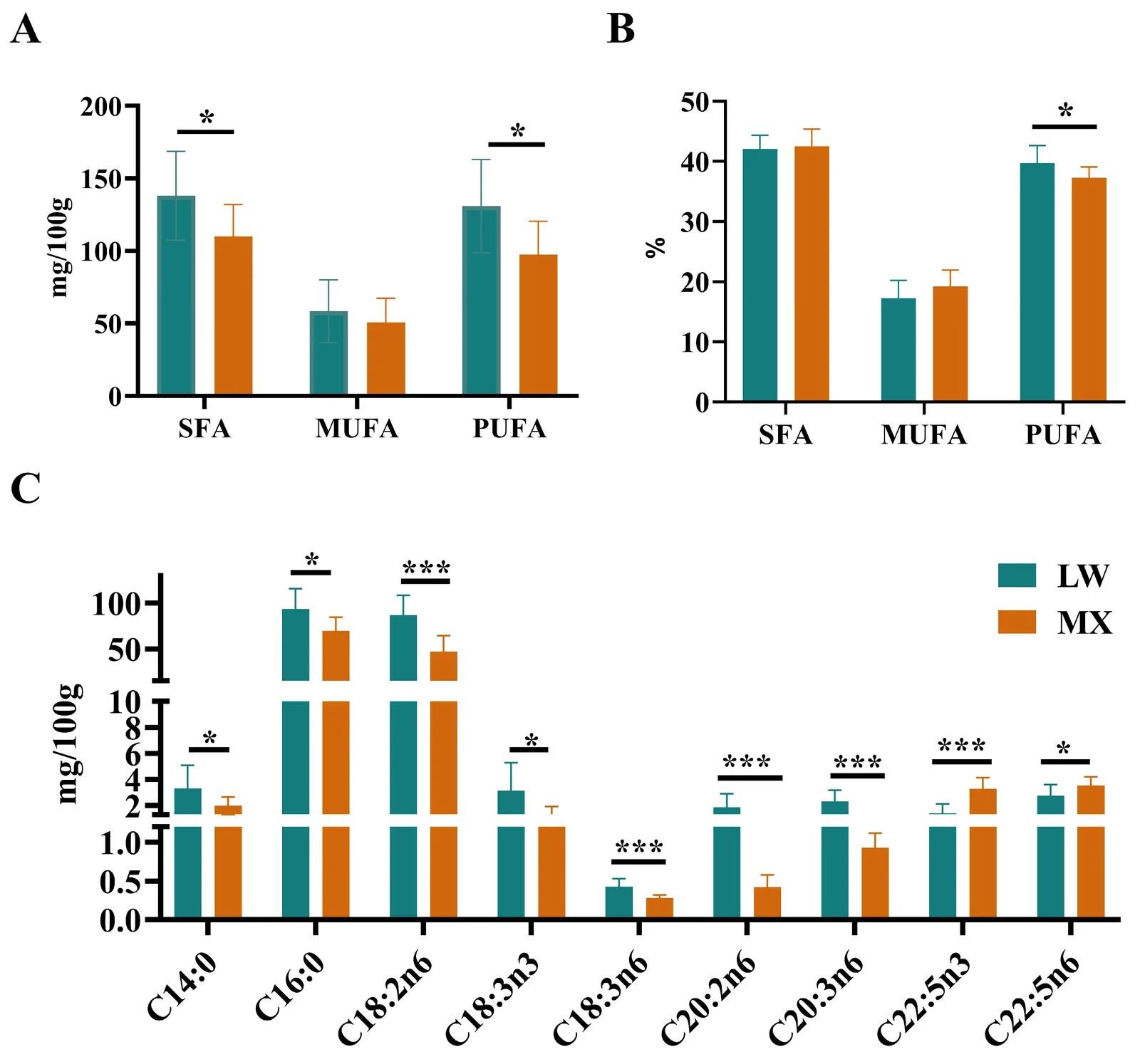
Fatty acid profile of longissimus dorsi muscle in Laiwu (LW) and Minxinan (MX) black rabbits. (**A**) Contents of saturated fatty acids (SFA), monounsaturated fatty acids (MUFA), and polyunsaturated fatty acids (PUFA) in total fatty acids (mg/100 g). (**B**) Proportions of saturated fatty acids (SFA), monounsaturated fatty acids (MUFA), and polyunsaturated fatty acids (PUFA) in total fatty acids. (**C**) Contents of individual fatty acids that differed significantly between breeds. Data are presented as mean ± SD (LW, *n* = 10; MX, *n* = 10). *, *p* < 0.05; ***, *p* < 0.001 (Student’s *t*-test). C14:0, myristic acid; C16:0, palmitic acid; C18:2n6, linoleic acid; C18:3n3, α-linolenic acid; C18:3n6, γ-linolenic acid; C20:2n6, eicosadienoic acid; C20:3n6, dihomo-γ-linolenic acid; C22:5n3, docosapentaenoic acid (n-3); C22:5n6, docosapentaenoic acid (n-6).

A total of 26 fatty acids were detected in the longissimus dorsi muscles of LW and MX rabbits, including 9 saturated fatty acids (SFA), 6 monounsaturated fatty acids (MUFA), and 11 polyunsaturated fatty acids (PUFA) (Table 1). Compared with MX rabbits, LW rabbits exhibited significantly higher total fatty acid content, with SFA and PUFA contents significantly elevated, while MUFA content did not differ significantly between breeds (Figure 1A). Regarding fatty acid proportions, the PUFA proportion in LW was significantly higher than that in MX (*p* < 0.05), whereas no significant differences were observed in SFA or MUFA proportions between the two breeds (*p* > 0.05) (Figure 1B).

At the individual fatty acid level, LW rabbits had significantly higher contents of C14:0 and C16:0 (end products of de novo fatty acid synthesis), as well as C18:2n6, C18:3n3, C18:3n6, C20:2n6, and C20:3n6, compared with MX rabbits; whereas MX rabbits had significantly higher contents of C22:5n3 and C22:5n6 than LW rabbits (Figure 1C). Moreover, the total n-6 PUFA content in LW was significantly higher than that in MX, while the n-6/n-3 ratio was significantly higher in LW than in MX. Taken together, the LD muscle of LW rabbits was characterized by greater overall fatty acid deposition, with SFA and PUFA increasing in parallel at the absolute level and a higher PUFA proportion at the compositional level.

### 3.2. Volatile Flavor Compositions Among Two Different Rabbit Breeds

A total of 448 VOCs were identified in the longissimus dorsi muscles of LW and MX rabbits based on reference spectra, the literature data, and retention time comparison. Among them, 194 compounds annotated by CAS numbers and with reported odor activity were selected as potential characteristic aroma contributors for detailed evaluation. These compounds were classified into nine groups, consisting of 29 aldehydes, 35 alcohols, 22 ketones, 23 esters, 22 hydrocarbons, 9 furans, 17 acids, 5 sulfides, and 32 others (Table S1).

**Figure 2:**
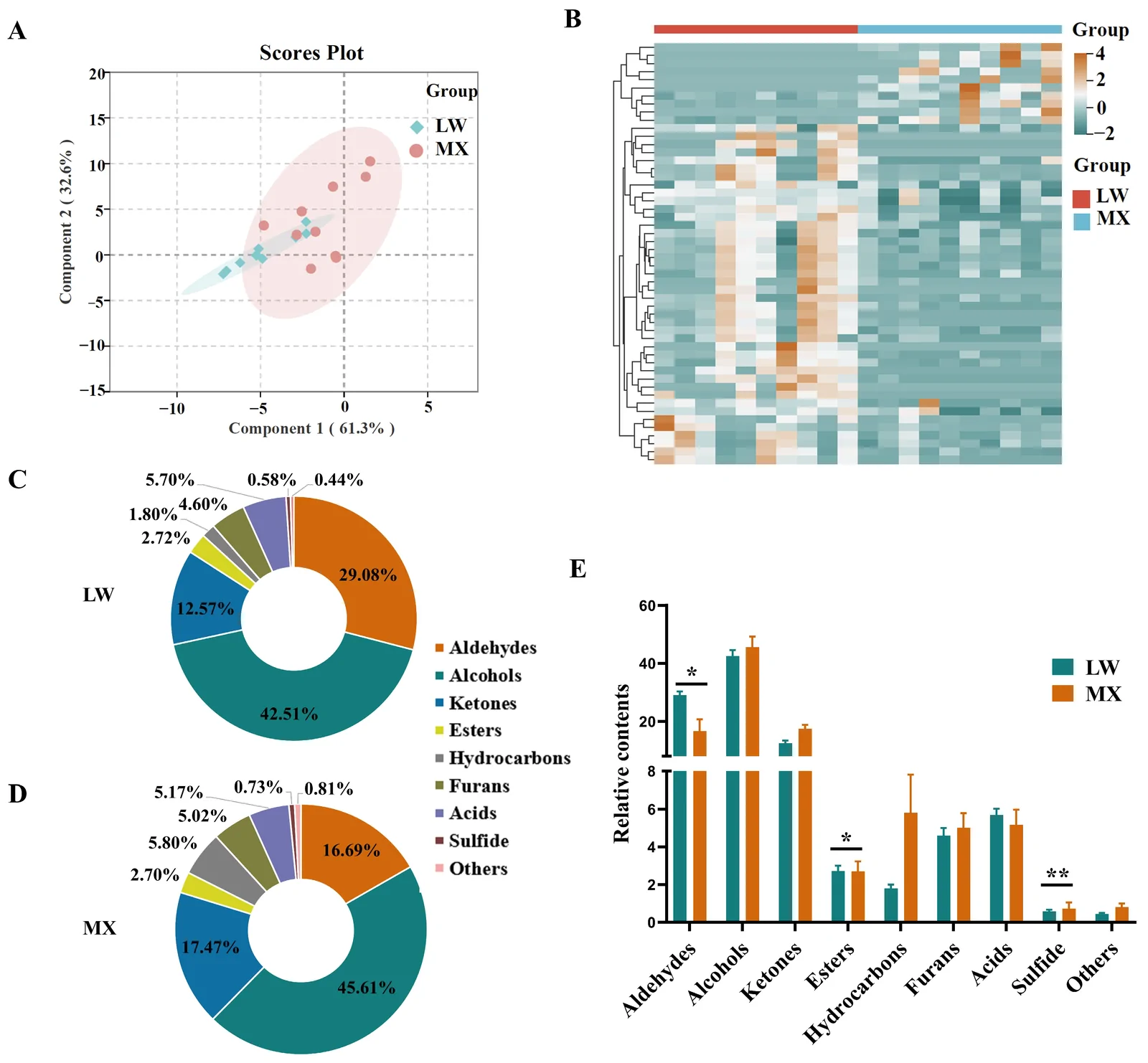
(**A**) PLS-DA score plot of VOCs. Shaded ellipses represent the 95% confidence ellipses of the LW and MX groups, respectively. (**B**) Heatmap of aroma compounds. (**C**,**D**) Percentages of aroma-compound categories in LW and MX. (**E**) Comparison of aroma-compound categories between LW and MX. *, *p* < 0.05; **, *p* < 0.01.

To distinguish the volatile profiles between breeds, partial least squares-discriminant analysis (PLS-DA) was applied, and the score plot showed clear separation between the two rabbit breeds (Figure 2A). The first two PLS-DA components explained 93.9% of the total variance, confirming discernible differences in the volatile profiles between LW and MX rabbits. Heatmap analysis further supported distinct clustering patterns between the two breeds (Figure 2B). The types and relative contents of aroma compounds are shown in Figure 2C,D, with alcohols constituting the predominant class in both breeds, accounting for 42.51% and 45.61% of the total aroma compounds in LW and MX rabbits, respectively. In LW rabbits, the proportions of aldehydes (29.08%) and esters (2.72%) were both significantly higher than those in MX rabbits, whereas the proportion of sulfides was significantly higher in MX rabbits than in LW rabbits (Figure 2E). A total of 52 compounds differed significantly between the two breeds (Table S2).

Of the 194 screened compounds, 104 had known odor thresholds, enabling calculation of relative odor activity values (ROAV) with 1-octen-3-ol as the reference compound. Among these, 25 compounds exhibited ROAV ≥ 0.1, of which 13 had ROAV ≥ 1, indicating key contributions to the overall aroma. By intersecting these 25 ROAV ≥ 0.1 compounds with the 52 differentially abundant compounds, 6 compounds were identified as both significantly different between breeds and with ROAV ≥ 0.1 (hexanal, ethanol, hexanoic acid, ethyl ester, pentanal, thiophene, 2,5-dimethyl-, and octanoic acid, ethyl ester) (Table 2).

### 3.3. Discrimination of Differential Metabolites and Functional Enrichment

Untargeted metabolomic profiling was performed on longissimus dorsi muscle samples from the two rabbit breeds. After exclusion of one outlier LW sample (LW10; quantitative per-breed Mahalanobis-distance criterion, Section 2.1 and Figure S1), a total of 19 samples (LW, *n* = 9; MX, *n* = 10) were subjected to LC-MS/MS analysis. Following removal of variables with a missing value rate > 20% or a relative standard deviation (RSD) > 30% in quality control (QC) samples, a total of 815 metabolites were retained for subsequent statistical analysis. Chemical classification of the 815 identified metabolites according to the HMDB database revealed that Lipids and lipid-like molecules constituted the largest proportion, accounting for 465 metabolites (57.06%), followed by Organic acids and derivatives (88 metabolites, 10.80%), Organoheterocyclic compounds (56 metabolites, 6.87%), and Organic oxygen compounds (47 metabolites, 5.77%). Benzenoids (36 metabolites, 4.42%), Nucleosides, nucleotides, and analogs (31 metabolites, 3.80%), and Organic nitrogen compounds (19 metabolites, 2.33%) were also detected at certain proportions.

**Figure 3:**
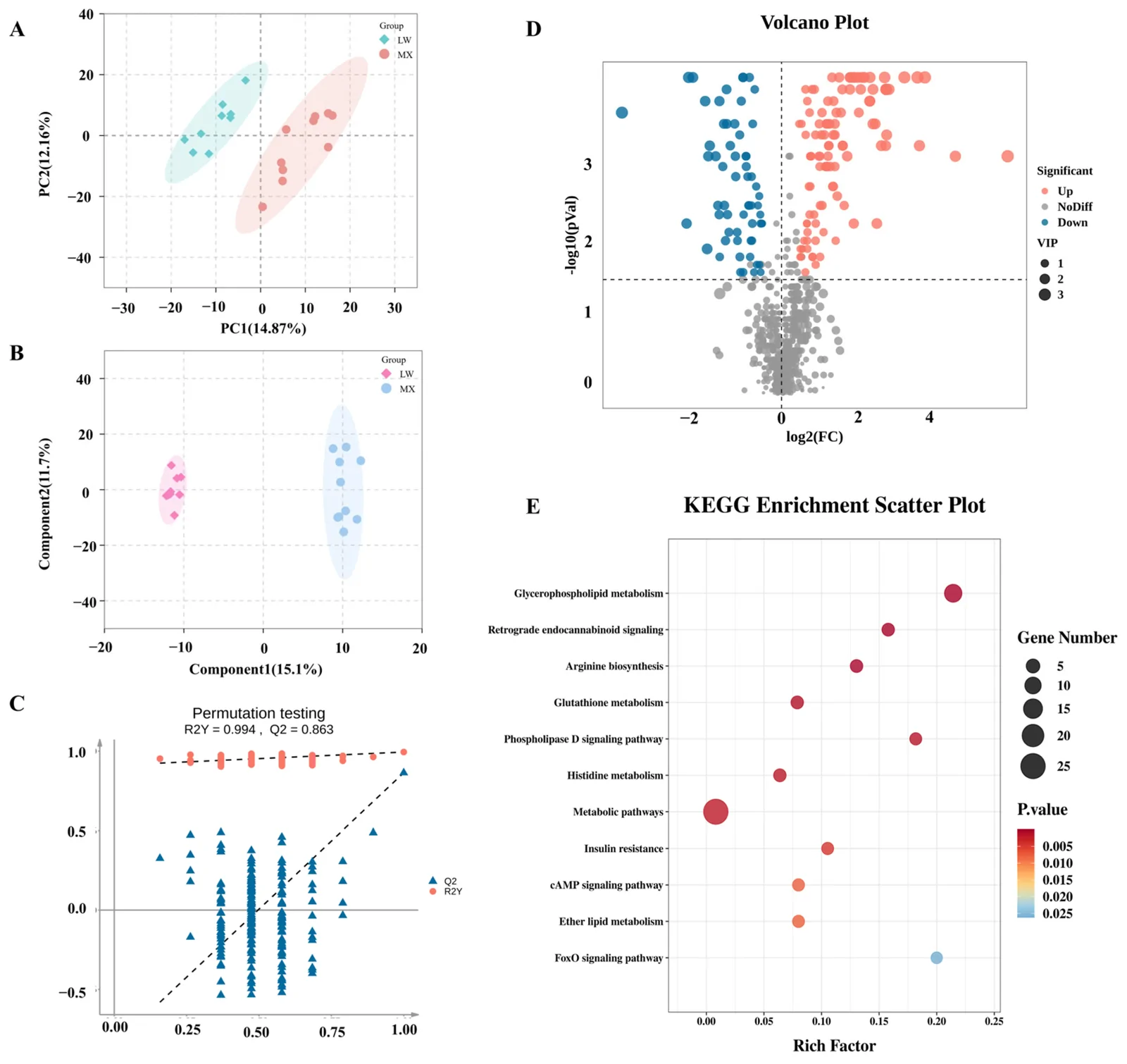
(**A**) PCA score plot of metabolic profiles. (**B**) OPLS-DA score plot of metabolic profiles. (**C**) 200-permutation test of the OPLS-DA model. Shaded ellipses represent the 95% confidence ellipses of the LW and MX groups, respectively. (**D**) Volcano plot of differentially abundant metabolites. (**E**) KEGG enrichment analysis of differentially abundant metabolites.

Principal component analysis (PCA) demonstrated a clear separation trend between the two groups (LW and MX) along the PC1 axis, indicating systematic differences in the metabolic profiles of the longissimus dorsi muscle between the two breeds (Figure 3A). PC1 explained 14.87% of the total variance, and PC2 explained 12.16%. Orthogonal partial least squares discriminant analysis (OPLS-DA) was employed to construct a discriminant model for the metabolic profiles of the LW and MX breeds (Figure 3B). The model exhibited a goodness of fit with R^2^X = 0.996 and R^2^Y = 0.996, and a predictive ability of Q^2^ = 0.901. A 200-permutation test confirmed that the model was not overfitted (R^2^ intercept = 0.943, Q^2^ intercept = −0.224; Figure 3C). Using VIP > 1 and *p* < 0.05 as selection criteria, a total of 102 differentially abundant metabolites were identified, of which 74 were upregulated and 28 were downregulated in the LW breed (Figure 3D). These differentially abundant metabolites were primarily enriched in Glycerophospholipid metabolism, Glutathione metabolism, Phospholipase D signaling pathway, and cAMP signaling pathway (Figure 3E).

### 3.4. DEGs Analysis and Functional Enrichment

**Figure 4:**
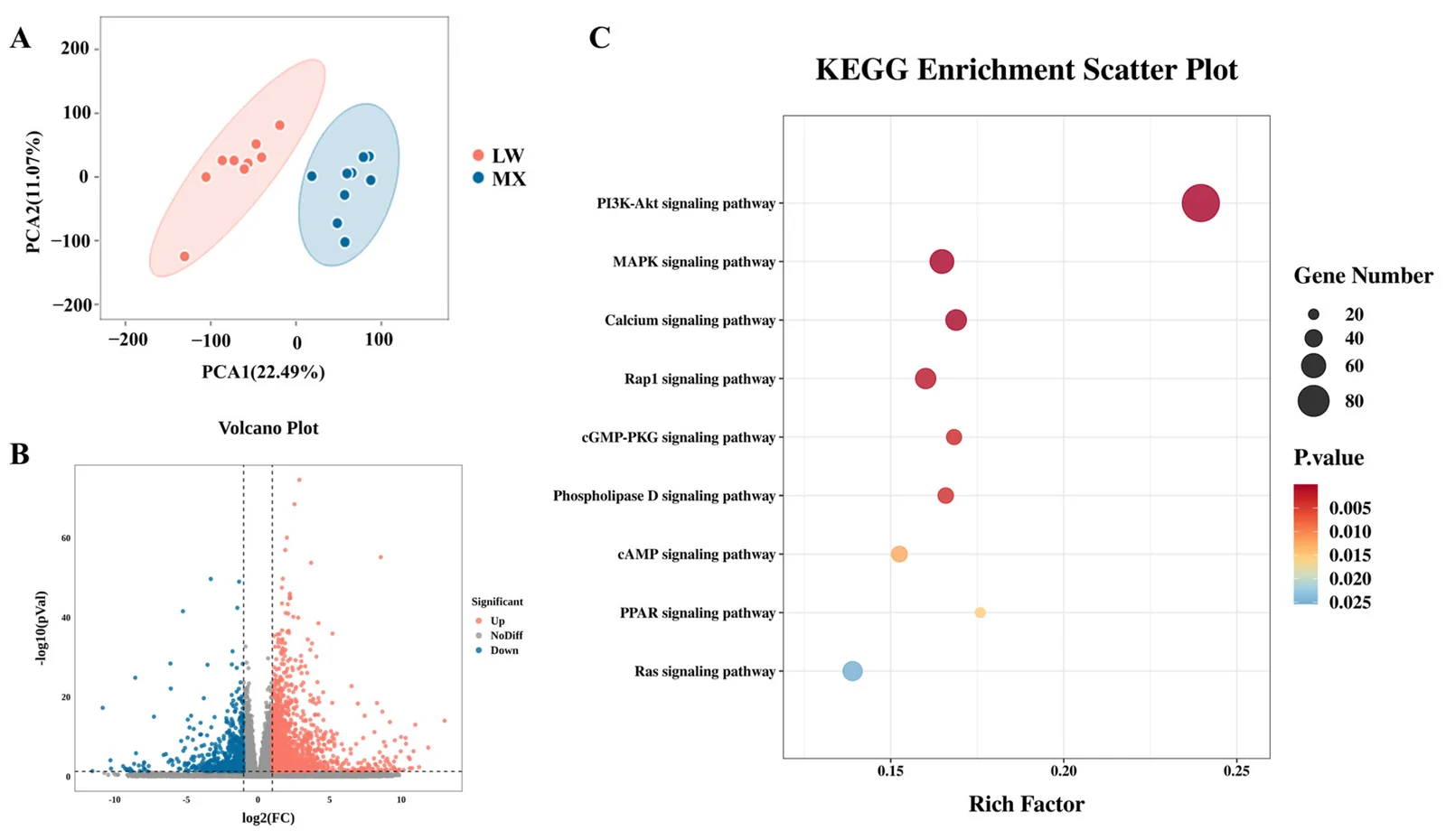
(**A**) PCA score plot of gene expression profiles in LW and MX rabbits. Shaded ellipses represent the 95% confidence ellipses of the LW and MX groups, respectively. (**B**) Volcano plot of differentially expressed genes (DEGs; |log_2_FC| > 1, FDR < 0.05). (**C**) KEGG pathway enrichment analysis of DEGs.

Transcriptome sequencing was performed on the longissimus dorsi (LD) muscle of the two rabbit breeds. After removing low-expression and non-informative genes, an expression matrix comprising 20 samples and 24,144 genes was established for subsequent differential expression analysis. Principal component analysis (PCA) of all expressed genes revealed that PC1 separated the two breeds, explaining 22.49% of the total variance (Figure 4A). The LW breed formed an independent cluster on the negative side of PC1, whereas the MX breed was located on the positive side. PC2 explained 11.07% of the variance, primarily reflecting intra-breed biological variation. The clear separation along PC1 indicates substantial interbreed differences in gene expression profiles. Using thresholds of |log_2_FC| > 1 and FDR < 0.05, a total of 2747 differentially expressed genes (DEGs) were identified between the LW and MX breeds. Among them, 2012 genes were upregulated and 735 were downregulated in LW relative to MX (Figure 4B). Functional annotation of the top 100 DEGs ranked by FDR revealed that genes associated with lipid metabolism included *DOC2B*, *SYT14*, *WDFY1*, *MARCKSL1*, *MEST*, *CCN5*, *SPARC*, *SPARCL1*, among others. KEGG pathway analysis identified 80 significantly enriched pathways (*p* < 0.05), of which lipid metabolism-related pathways included the PPAR signaling pathway, Phospholipase D signaling pathway, cAMP signaling pathway, and MAPK signaling pathway (Figure 4C).

### 3.5. Integrated Transcriptomic–Metabolomic Analysis

#### 3.5.1. Two-Way Orthogonal Partial Least Squares (O2PLS) Modeling

An O2PLS model integrating 1139 KEGG-annotated DEGs (X-block) and 49 KEGG-mapped DAMs (Y-block) identified two joint components, explaining 59.6% of transcriptomic variance (R^2^X) and 67.5% of metabolomic variance (R^2^Y), with a correlated fraction R^2^Ycor of 58.0%. This substantial covariance supports the validity of a joint analysis framework for discriminating the two breeds.

**Figure 5:**
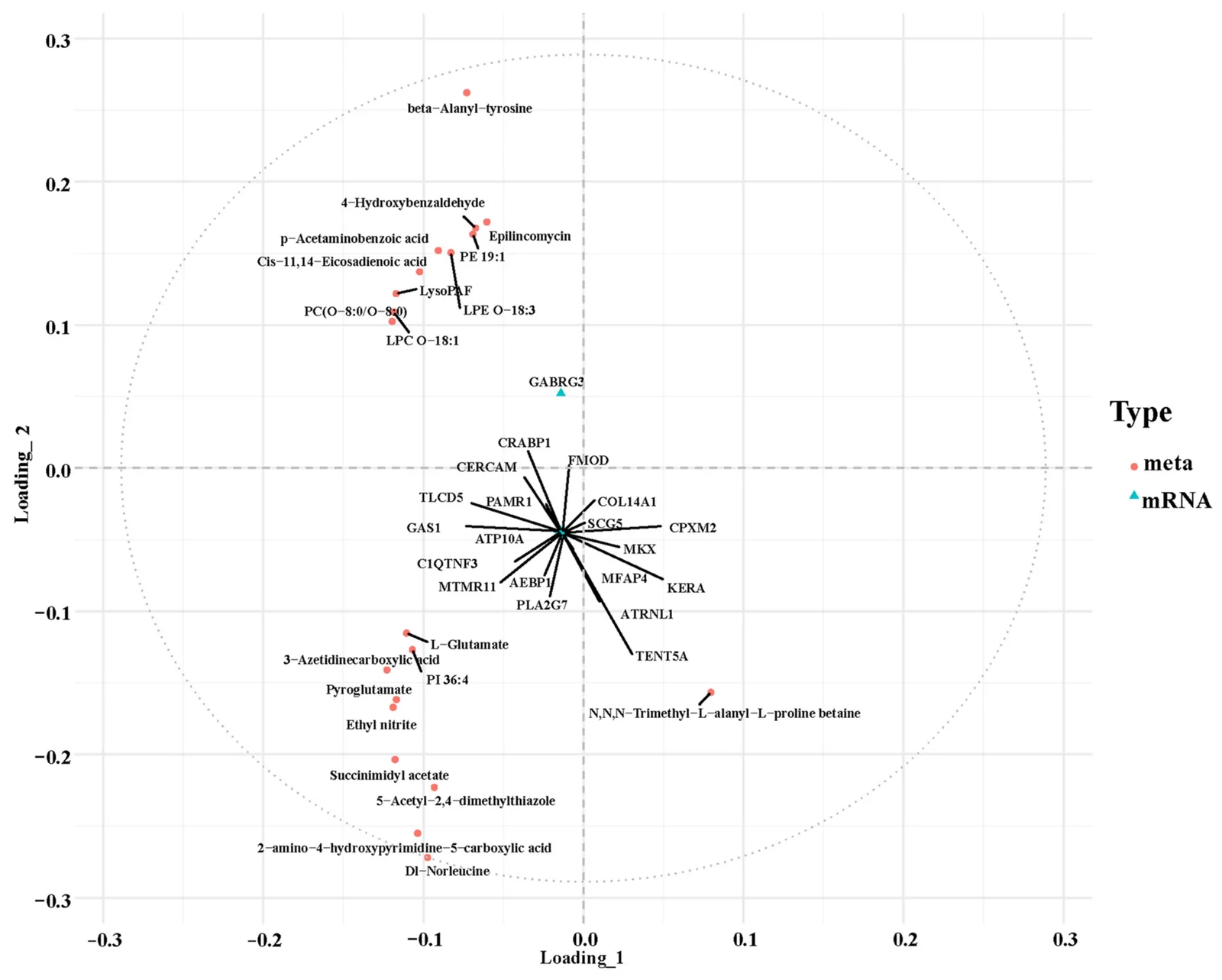
Joint O2PLS loading plot of transcriptome and metabolome discriminating LW and MX rabbits.

Top mRNA contributors (by squared loading on joint component 1) included *GABRG3*, *PLA2G7*, *TENT5A*, *AEBP1*, and *MFAP4*, while top discriminant metabolites were dominated by amino acid derivatives (e.g., DL-Norleucine, Pyroglutamate) and the sulfur-containing heterocycle 5-acetyl-2,4-dimethylthiazole. This pattern indicates a tight coupling between breed-discriminatory transcriptomic architecture and amino acid/volatile precursor metabolism (Figure 5).

#### 3.5.2. Pearson Correlation Network

A pairwise Pearson correlation analysis of the 2761 differentially expressed genes (DEGs) and 102 differentially abundant metabolites (DAMs) generated 281,622 gene–metabolite pairs. Of these, 161,248 (57.3%) exhibited significant correlations (*p* < 0.05), comprising 103,947 positive and 57,301 negative associations. These correlations remained significant under more stringent statistical thresholds (92,972 pairs at *p* < 0.01; 36,878 at *p* < 0.001). The predominance of positive correlations (64.5% of all significant pairs) suggests that transcriptional upregulation generally aligns with increased metabolite abundance.

**Figure 6:**
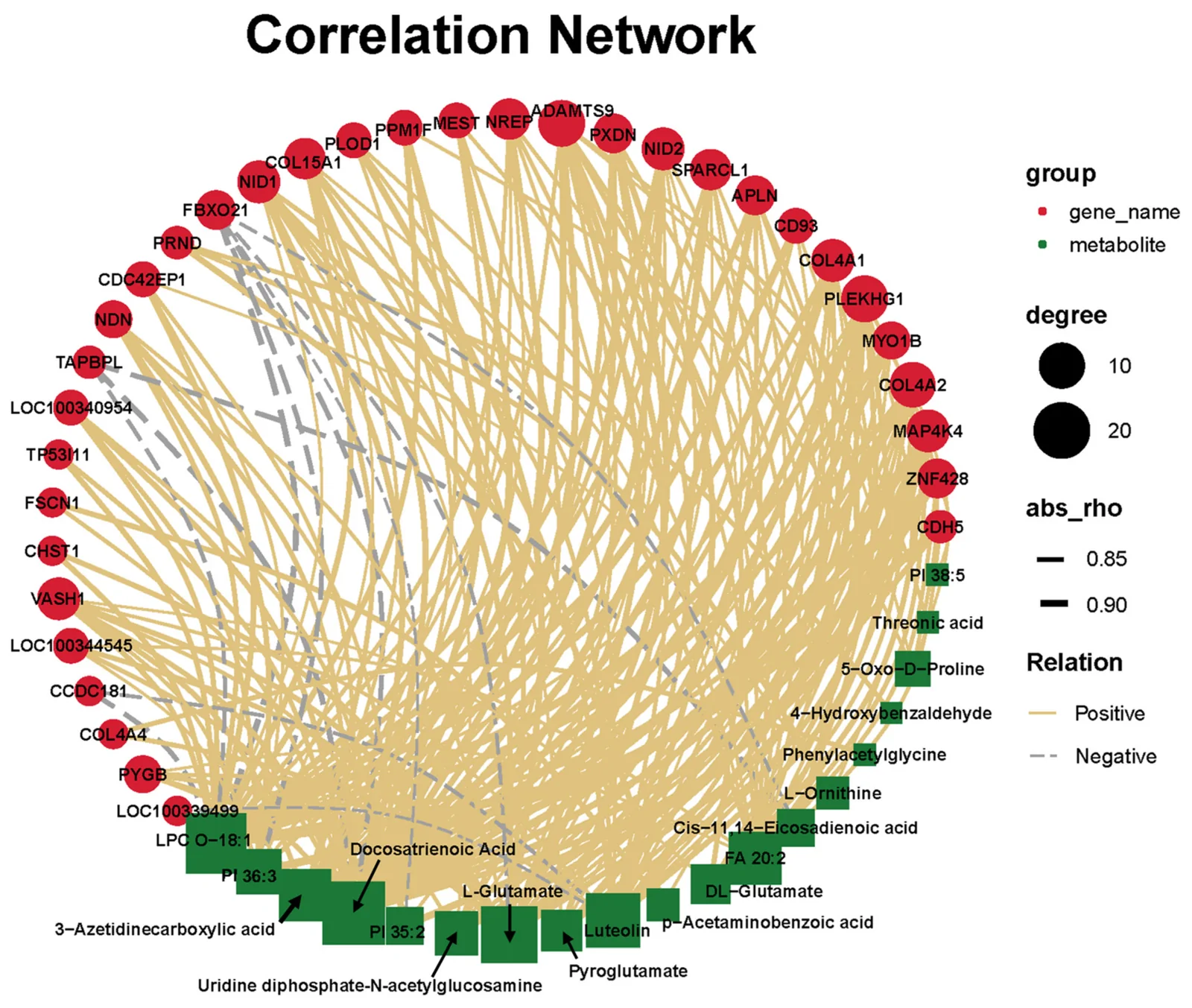
Correlation network of top 35 transcriptomic and metabolomic features between LW and MX rabbit breeds (|r| > 0.8, *p* < 0.05).

Notable strong correlations (|r| > 0.95) included *MATCAP1* with two ether-linked lysophospholipids (e.g., LPC O-16:0, r = 0.962) and *FGFR4* with argininic acid (r = 0.962), suggesting a close association between membrane lipid remodeling and arginine catabolism. Hub analysis identified *ZNF428*, *LAMB1*, *FSCN1*, *ECT2*, and *DTX4* as top hub genes, and argininic acid, edoxudine, docosatrienoic acid (C22:3), and methylhistidine isoforms as top hub metabolites (each with >2200 connections), reinforcing protein/amino acid turnover and lipid metabolism as dominant axes of inter-breed variation (Figure 6, Table S3).

#### 3.5.3. Common KEGG Pathway Enrichment

**Figure 7:**
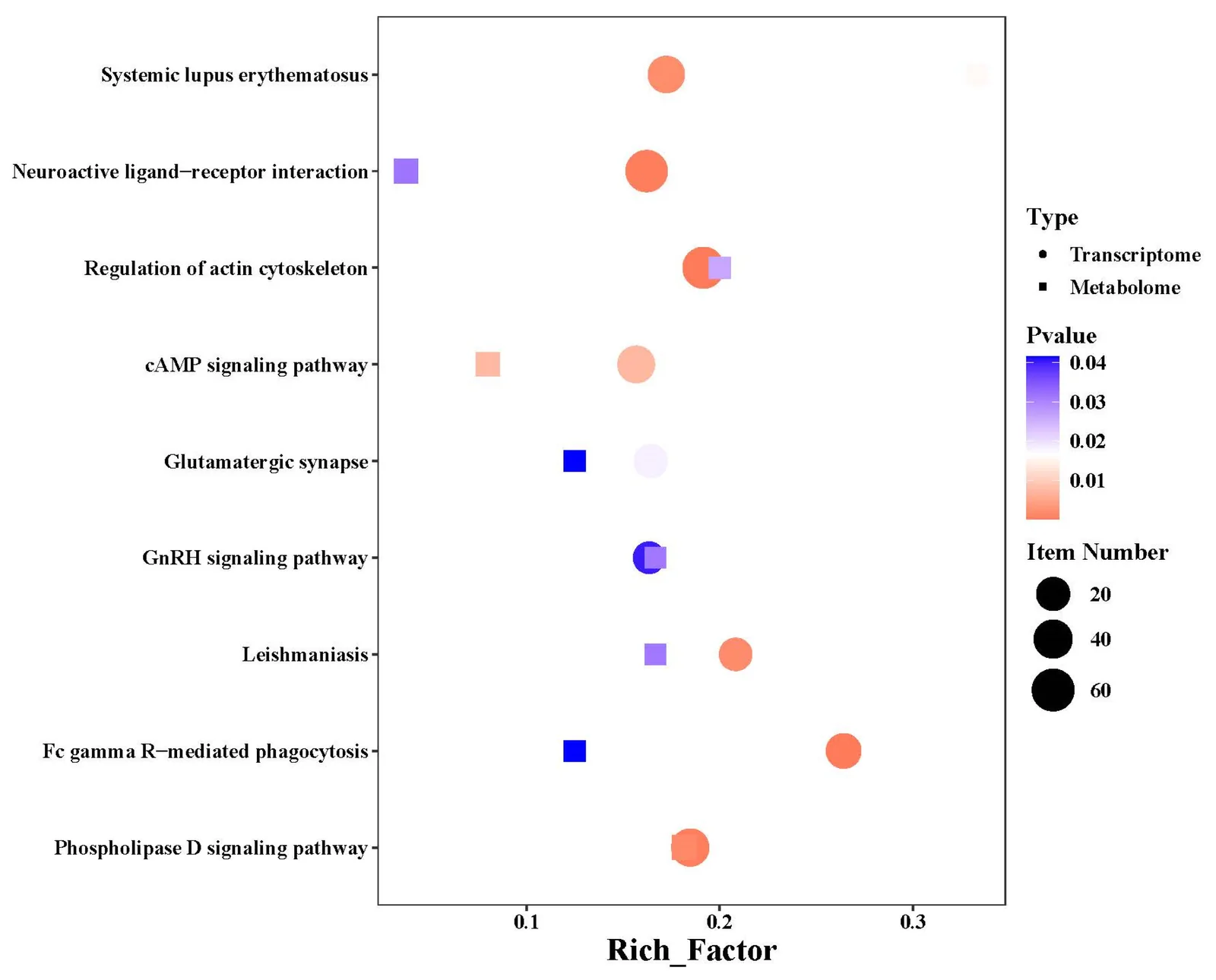
Co-enriched KEGG pathways of differentially expressed genes (DEGs) and differentially abundant metabolites (DAMs) in the longissimus dorsi muscle between Laiwu Black (LW) and Minxinan Black (MX) rabbits. Dot plot showing the ten KEGG pathways significantly enriched (*p* < 0.05) in both the DEG and DAM analyses. Each pathway is represented by two data points: a circle (transcriptome) and a square (metabolome). The *x*-axis indicates the Rich Factor (the ratio of DEGs or DAMs mapped to a pathway to the total number of DEGs or DAMs annotated in the KEGG database). Dot color reflects the enrichment *p* value (blue, *p* = 0.04; orange, *p* = 0.01), and dot size represents the number of DEGs or DAMs mapped to each pathway.

Independent KEGG enrichment of DEGs and DAMs identified ten co-enriched pathways meeting *p* < 0.05 in both analyses: Regulation of actin cytoskeleton, Fc gamma R-mediated phagocytosis, Phospholipase D signaling, Neuroactive ligand–receptor interaction, Systemic lupus erythematosus, Leishmaniasis, cAMP signaling, Glutamatergic synapse, Kaposi sarcoma-associated herpesvirus infection, and GnRH signaling (Figure 7, Table S4). Functionally, these pathways converged into three biological themes. First, the Phospholipase D and cAMP signaling pathways jointly implicated lipid signaling and membrane remodeling, involving dysregulated PLD2 (encoding the principal PLD isoform) and multiple LPAR receptors, alongside the shared metabolite PA (16:0/18:2). Second, the Regulation of actin cytoskeleton pathway reflected ECM–cytoskeleton remodeling, involving LAMB1, collagens (*COL4A1*, *COL3A1*), and the RhoGEF ECT2. Third, the Neuroactive ligand–receptor interaction, Glutamatergic synapse, and GnRH signaling pathways converged on neuroactive signaling, suggesting that neuromuscular junction activity and hormonal signaling may also contribute to the metabolic divergence in longissimus dorsi between LW and MX rabbits.

#### 3.5.4. Convergent Lipid Signaling Module

Converging lines of evidence from O2PLS, Pearson correlation, and KEGG analyses consistently highlighted the PLD–PA–LPAR lipid signaling axis as a key molecular differentiator between the two breeds. Specifically, *PLA2G7* (a top contributor in O2PLS analysis associated with glycerophospholipid metabolism), PLD2/LPAR (driving enrichment of the Phospholipase D pathway), and ether-linked lysophospholipids (strongly correlated with hub genes) collectively converged on this axis. Given the distinct fatty acid compositions between breeds, the coordinated transcriptional regulation of this pathway in LW rabbits may prioritize PUFA-rich triacylglycerol synthesis over phospholipid turnover, thereby shaping breed-specific flavor profiles. Consistent with this convergent picture, the key genes of the PLD–PA–LPAR axis were differentially expressed in LW relative to MX (DESeq2, *n* = 10 LW and 10 MX; Table S5): *LPAR1* (log_2_FC = 1.11, FDR = 5.79 × 10^−4^), *LPAR2* (log_2_FC = 2.27, FDR = 9.04 × 10^−4^), and *LPAR4* (log_2_FC = 2.55, FDR = 8.37 × 10^−16^) were significantly up-regulated in LW, exceeding both the FDR threshold and a |log_2_FC| ≥ 1 fold-change criterion. *PLA2G7* (log_2_FC = 0.95, FDR = 1.65 × 10^−2^) and *PLD2* (log_2_FC = 0.97, FDR = 2.63 × 10^−7^) also showed FDR-significant up-regulation in LW, although their fold changes were more modest (<1.1). By contrast, *LPAR3* (log_2_FC = 1.53, FDR = 0.46), *LPAR5* (log_2_FC = −0.71, FDR = 0.57), and *LPAR6* (log_2_FC = 0.00, FDR = 0.98) did not reach significance. The explicit direction and magnitude of these changes make the PLD–PA–LPAR narrative directly checkable from the differential-expression results rather than inferred from loading or network plots.

### 3.6. RT-qPCR Validation of Key Differentially Expressed Genes

To validate the transcriptomic findings, six key genes involved in the PLD–PA–LPAR axis and lipid metabolism (*PLA2G7*, *PLD2*, *LPAR1*, *LPAR4*, *ELOVL5*, and *DGAT2*) were selected for RT-qPCR validation using the same LD muscle samples (*n* = 10 per breed). The direction of differential expression was concordant with the RNA-seq data for all six genes (Figure S3, Table S6). Significant inter-breed differences were confirmed for all six genes (two-tailed Welch’s *t*-test, *p* < 0.05 for each): *PLD2* (LW 1.12 ± 0.48 vs. MX 0.46 ± 0.26; 2.44-fold, *p* = 0.0019), *PLA2G7* (LW 1.18 ± 0.56 vs. MX 0.55 ± 0.20; 2.14-fold, *p* = 0.0061), *LPAR1* (LW 1.14 ± 0.64 vs. MX 0.61 ± 0.23; 1.86-fold, *p* = 0.033), *LPAR4* (LW 1.10 ± 0.42 vs. MX 0.33 ± 0.17; 3.30-fold, *p* < 0.0001), *ELOVL5* (LW 1.04 ± 0.17 vs. MX 0.54 ± 0.23; 1.92-fold, *p* < 0.0001), and *DGAT2* (LW 0.83 ± 0.33 vs. MX 2.01 ± 0.57; 2.42-fold higher in MX, *p* < 0.0001). The RT-qPCR and RNA-seq log2 fold-changes were strongly positively correlated across the six genes (Pearson r = 0.95, R^2^ = 0.91, *p* = 0.003).

## 4. Discussion

This study shows distinct intramuscular fatty acid profiles between LW and MX rabbits. LW rabbits exhibited significantly higher total fatty acid content, primarily driven by elevated saturated fatty acids (SFAs) and polyunsaturated fatty acids (PUFAs), while monounsaturated fatty acid (MUFA) levels remained comparable between breeds. Among individual fatty acids, the most pronounced breed differences were observed in C14:0 (myristic acid) and C16:0 (palmitic acid)—the two canonical end products of de novo fatty acid synthesis catalyzed by fatty acid synthase (FASN) [20,21,22]. The coordinated upregulation of these de novo synthesis markers in LW rabbits suggests a breed-intrinsic difference in lipogenic capacity within the longissimus dorsi muscle, consistent with transcriptomic evidence of PPAR signaling pathway enrichment in this breed.

The higher levels of C18:2n6 (linoleic acid) and C18:3n3 (α-linolenic acid) in LW rabbits are particularly significant, as these essential fatty acids cannot be synthesized de novo by mammals and must be obtained through the diet. Given that both breeds were raised under identical feeding conditions, the elevated content of these essential PUFAs in LW muscle likely reflect enhanced selective uptake and incorporation of dietary PUFA into muscle phospholipids and triacylglycerols rather than differences in dietary intake. This interpretation is supported by the O2PLS model, which identified *PLA2G7*—a gene encoding a phospholipase A2 involved in hydrolyzing oxidized phospholipids and participating in glycerophospholipid metabolism [23]—as a top loading gene distinguishing the two breeds. The higher total n-6 PUFA content and n-6/n-3 ratio in LW rabbits may therefore reflect breed-specific differences in phospholipid remodeling and membrane fatty acid composition. Alternative or additional mechanisms should also be considered, however: breed differences in lipid turnover rate, oxidative loss of PUFA, the extent of adipocyte differentiation, or overall intramuscular fat deposition capacity could each contribute to the observed PUFA pattern independently of transport-protein selectivity. The present data cannot disentangle these alternatives, and the selective-uptake interpretation should therefore be regarded as one of several plausible explanations rather than the sole mechanism.

Conversely, MX rabbits exhibited higher concentrations of C22:5n3 (DPA) and C22:5n6, which are elongation and desaturation products derived from C18:3n3 (α-linolenic acid) and C18:2n6 (linoleic acid), respectively [24]. The inverse relationship between precursor PUFA (C18:2n6, C18:3n3; LW > MX) and their elongation products (C22:5n6, C22:5n3; MX > LW) suggests that MX rabbits possess higher elongation activity, potentially mediated by ELOVL2/5 elongases. This breed-specific elongation phenotype may represent an adaptive trait in commercial breeds selected for rapid growth, consistent with previous reports that commercial rabbit breeds exhibit distinct intramuscular fatty acid profiles compared with local or hybrid breeds [1,25].

The PLS-DA score plot demonstrated clear separation of volatile organic compound (VOC) profiles between LW and MX breeds, with the first two components explaining 93.9% of total variance, indicating that breed is a dominant determinant of meat aroma composition. Alcohols constituted the predominant aroma class in both breeds (>42%), in contrast to previous studies in which aldehydes have been reported as the most abundant VOC class in rabbit meat [26,27]. However, LW rabbits exhibited significantly higher proportions of aldehydes (29.08%) and esters (2.72%) compared with MX rabbits, whereas MX rabbits showed a higher proportion of sulfides.

A strength of this study is the integration of ROAV-based odorant screening with differential abundance analysis. Among the 52 differentially abundant compounds and 25 compounds with ROAV ≥ 0.1, six key odorants were identified: hexanal, ethanol, ethyl hexanoate, pentanal, 2,5-dimethylthiophene, and ethyl octanoate. Hexanal, derived from the oxidation of linoleic acid (C18:2n6), is widely recognized as a major volatile marker and key aroma contributor in meat products [5,28]. The higher hexanal content in LW rabbits aligns mechanistically with their elevated C18:2n6 levels (Section 3.1), reinforcing the sequential pathway from fatty acid substrates via lipid oxidation to flavor compound biosynthesis.

Ethyl hexanoate and ethyl octanoate impart fruity and floral notes, whereas 2,5-dimethylthiophene, a sulfur-containing heterocyclic compound, likely arises from Maillard reactions involving cysteine or methionine [4]. The higher proportion of sulfur-containing compounds in MX rabbits may reflect breed-specific differences in free amino acid pools or thermal degradation pathways during cooking. Notably, the predominance of lipid-derived compounds (hexanal, pentanal, ethyl esters) as the main discriminative odorants highlights the central role of intramuscular lipid composition in defining breed-specific meat flavor characteristics.

Untargeted metabolomics analysis revealed that lipids and lipid like molecules constituted the dominant metabolite class (57.06% of all detected metabolites), consistent with skeletal muscle’s specialized role in lipid storage and metabolism [29]. OPLS-DA modeling demonstrated strong predictive performance (Q^2^ = 0.901), confirming robust metabolic divergence between LW and MX breeds. Of the 102 differentially abundant metabolites (DAMs), enrichment analysis highlighted significant perturbations in glycerophospholipid metabolism and phospholipase D signaling pathways.

Transcriptomic analysis identified 2747 differentially expressed genes (DEGs) across breeds, with KEGG enrichment highlighting PPAR signaling, phospholipase D signaling, and cAMP signaling pathways closely associated with lipid metabolism. The enrichment of the PPAR signaling pathway in LW rabbits is mechanistically important. Although PPARD itself was not among the differentially expressed genes, several enriched members of this pathway—most notably *FABP5* (fatty acid-binding protein 5, a canonical PPARδ target that shuttles fatty acids to the nucleus for PPARδ transactivation), *CPT1A* (carnitine palmitoyltransferase 1A, the rate-limiting enzyme of mitochondrial fatty acid β-oxidation), *ANGPTL4* (angiopoietin-like 4, a PPARδ-regulated inhibitor of lipoprotein lipase that modulates tissue lipid uptake), and *SLC27A2* (solute carrier family 27 member 2, a long-chain fatty acid transporter)—are well established transcriptional targets of PPARδ in skeletal muscle [30,31], collectively consistent with enhanced PPARδ transcriptional activity in LW longissimus dorsi. PPARδ is the predominant PPAR isoform in skeletal muscle and serves as a master transcriptional regulator of fatty acid uptake, β-oxidation, and oxidative fiber-type specification [30,31]. Activation of PPARδ has been shown to increase intramuscular PUFA content and shift muscle metabolism toward lipid utilization [32], suggesting a possible transcriptional mechanism for the elevated PUFA phenotype observed in LW rabbits. Direct measurements of muscle fiber-type composition are not available for the LW and MX breeds used here, and this inference should therefore be tested in future work.

An important finding of this study is the multimethod approach that identified the PLD–PA–LPAR signaling axis as a central molecular discriminator between LW and MX longissimus dorsi muscles. This conclusion was supported by three independent analytical frameworks: (1) *PLA2G7* emerged as a top-ranking gene in O2PLS analysis, demonstrating strong association with lipid metabolism; (2) PLD2 and LPAR family members drove phospholipase D signaling pathway enrichment in KEGG analysis; and (3) ether-linked lysophospholipids (e.g., PC(O-8:0/O-8:0), LPC O-16:0) exhibited extremely high correlations (r > 0.96) with *MATCAP1* in the Pearson network.

Phospholipase D (PLD) catalyzes the hydrolysis of phosphatidylcholine to produce phosphatidic acid (PA), which serves dual roles: as a lipid second messenger that activates downstream signaling cascades and as a metabolic precursor for diacylglycerol (DAG) and triacylglycerol (TAG) synthesis [33,34]. This positions PLD at a critical intersection between phospholipid remodeling and neutral lipid storage. The coordinated up-regulation of PLD2 and several LPAR family members (LPAR1, LPAR2, LPAR4) in LW rabbits (Section 3.5.4; Table S5), combined with elevated intramuscular PUFA content, indicates breed-specific enhancement of the PLD–PA–LPAR signaling axis; enhanced PLD-derived PA flux may therefore favor channeling of fatty acyl-CoA toward PUFA-enriched TAG deposition and contribute to the higher intramuscular fat content of this breed. Additionally, lysophosphatidic acid (LPA), generated from PA via phospholipase A-mediated deacylation, exerts pleiotropic effects on cell differentiation through six cognate LPAR receptors [35,36]; the up-regulation of multiple LPAR family members in LW rabbits may strengthen LPA-mediated signaling, thereby contributing to distinct intramuscular fat accumulation patterns between the two breeds. The precise causal relationship between PLD–PA–LPAR activation and lipid partitioning warrants functional validation.

These transcriptomic signatures were further supported by independent RT-qPCR validation of six key genes, which reproduced the direction of all observed changes (Section 3.6).

The integrated multi-omics approach presented in this study supports a model in which breed-specific differences in rabbit longissimus dorsi meat quality stem from two interrelated molecular networks.

Activation of the breed-intrinsic PPAR signaling pathway (particularly PPARδ) enhances de novo lipogenesis, as evidenced by elevated C14:0 and C16:0 levels, while simultaneously promoting the selective incorporation of dietary essential fatty acids C18:2n6 and C18:3n3 into muscle tissue. These coordinated changes expand the intramuscular linoleic acid substrate pool, which during cooking undergoes lipid oxidation to generate higher levels of hexanal and other aldehyde-derived key odorants, producing the breed-characteristic meat aroma.

Breed-specific differences in PLD–PA–LPAR signaling activity are associated with the allocation of fatty acyl-CoA between phospholipid turnover and triacylglycerol (TAG) storage. This differential partitioning reshapes membrane lipid composition, including ether-linked phospholipid content, and modulates intramuscular fat deposition, potentially contributing to interbreed differences in meat quality traits such as juiciness, tenderness, and flavor retention.

The interplay between these two molecular networks is linked at the level of fatty acid substrate allocation: PPAR signaling enhances the overall flux of fatty acids into muscle tissue, while the strength of PLD signaling is associated with the proportion of this flux directed toward structural phospholipids versus triacylglycerol (TAG) storage. This model provides a systems-level framework for understanding breed-specific meat quality determination. Consistent with this framework, Kuang et al. [9] recently reported that lipid metabolism and extracellular matrix (ECM)-related pathways serve as key discriminators between Shuxing No. 1 and IRA rabbit breeds using a similar multi-omics approach, while Liu et al. [10] demonstrated that the interaction between cooking methods and breed significantly influences volatile flavor profiles in rabbit meat.

Four limitations should be acknowledged. First, the relatively small sample size (LW: *n* = 9–10; MX: *n* = 10), while sufficient for detecting main breed effects, may limit statistical power to identify subtle breed × environment interactions. Second, molecular associations identified through O2PLS and Pearson correlation analyses, although robust across independent modules, do not establish causality. Experimental validation, including qRT-PCR confirmation of key differentially expressed genes (DEGs) and functional perturbation of the PLD–PA–LPAR axis in myoblast models, is required to confirm the proposed gene–lipid–volatile causal chain. Third, conventional meat quality parameters (pH, instrumental color, water holding capacity, shear force) and carcass composition, body weight, and growth rate traits were not measured concurrently with the omics profiling in the present cohort, and breed-level differences in overall fatness could influence the lipidomic and transcriptomic signatures reported here. To ground our molecular findings in established breed phenotypes, we refer to our team’s independent characterization of the same two breeds, sourced from the same breeding company and reared under identical husbandry [37], which reported significant breed divergence in precisely these traits: shear force (LW 26.3 N vs. MX 31.0 N, *p* < 0.05), drip loss (LW 1.72% vs. MX 2.92%, *p* < 0.05), intramuscular fat (LW 2.58% vs. MX 1.70%, *p* < 0.01), and pre-slaughter body weight (LW 2449 g vs. MX 1558 g, *p* < 0.001). These independently demonstrated breed differences confirm that the fatty-acid, volatile, and transcriptomic signatures reported here coincide with genuine, consumer-relevant meat-quality divergence between LW and MX, although matched phenotyping of the omics cohort remains a clear direction for future work. Fourth, flavor-related molecular characterization relied on theoretically computed ROAVs without parallel sensory-panel evaluation; moreover, the odor thresholds used for OAV/ROAV calculation are typically determined in water or air rather than in a meat/lipid matrix, and matrix effects can substantially influence perceived thresholds and therefore the ROAV estimates. Future studies integrating sensory analysis with comprehensive meat-quality phenotyping alongside multi-omics profiling will be essential to translate molecular signatures into consumer-relevant quality attributes.

## 5. Conclusions

This study integrated volatilomic, metabolomic, and transcriptomic profiling to characterize breed-specific meat flavor differences between LW and MX rabbits. LW rabbits exhibited higher intramuscular fatty acid content, particularly de novo synthesis markers (C14:0, C16:0) and essential PUFAs (C18:2n6, C18:3n3), while MX rabbits showed elevated elongation products (C22:5n3, C22:5n6). ROAV analysis identified hexanal—derived from C18:2n6 oxidation—as the most breed-discriminating key odorant among six identified volatiles. Multi-omics integration through O2PLS, Pearson correlation, and KEGG co-enrichment convergently identified the PLD–PA–LPAR lipid signaling axis as a central molecular discriminator, with PLA2G7, PLD2, and LPAR members consistently prioritized across all three modules. The integrated model indicates that PPARδ-driven lipogenesis supplies PUFA substrates for hexanal generation, while PLD–PA–LPAR signaling is associated with fatty acyl-CoA allocation between membrane phospholipid turnover and triacylglycerol storage, together associated with breed-characteristic rabbit meat flavor. These findings suggest potential molecular targets for genetic selection or nutritional strategies to improve rabbit meat quality, pending functional validation of the putative mechanism linking PLD–PA–LPAR signaling to PUFA synthesis and hexanal formation.

## Figures and Tables

**Table 1:** Meat Fatty Acid Composition (mg/100 g) of MX rabbit and LW rabbit.

	LW	MX	*p*-Value
Saturated fatty acids (SFA)			
C10:0	1.39 ± 1.25	0.72 ± 0.44	0.130
C14:0	3.31 ± 1.76	1.99 ± 0.67	0.040
C15:0	1.84 ± 0.59	1.46 ± 0.30	0.086
C16:0	93.65 ± 22.19	69.87 ± 14.62	0.011
C17:0	1.75 ± 0.54	1.53 ± 0.36	0.299
C18:0	35.63 ± 6.47	33.67 ± 6.01	0.492
C20:0	0.43 ± 0.08	0.43 ± 0.07	0.988
C22:0	0.19 ± 0.03	0.18 ± 0.03	0.665
C24:0	0.19 ± 0.04	0.21 ± 0.08	0.397
Monounsaturated fatty acids (MUFA)			
C15:1n5	1.04 ± 0.63	1.08 ± 0.70	0.905
C16:1n7	3.30 ± 1.80	2.57 ± 1.11	0.287
C18:1n7	4.81 ± 2.09	4.14 ± 1.38	0.413
C18:1T	0.43 ± 0.08	0.47 ± 0.06	0.286
C18:1n9	51.14 ± 19.16	44.14 ± 14.95	0.374
C20:1n9	1.15 ± 0.42	0.95 ± 0.21	0.178
Polyunsaturated fatty acids (PUFA)			
C18:2n6	86.83 ± 21.6	47.12 ± 17.3	<0.001
C18:3n3	3.13 ± 2.14	1.32 ± 0.59	0.019
C18:3n6	0.43 ± 0.10	0.28 ± 0.04	<0.001
C20:2n6	1.88 ± 1.03	0.42 ± 0.16	<0.001
C20:3n6	2.31 ± 0.86	0.93 ± 0.19	<0.001
C20:4n6	20.68 ± 11.59	28.88 ± 5.51	0.058
C20:5n3	0.36 ± 0.08	0.38 ± 0.07	0.505
C22:4n6	3.88 ± 1.44	3.16 ± 0.77	0.177
C22:5n3	1.39 ± 0.72	3.28 ± 0.85	<0.001
C22:5n6	2.75 ± 0.85	3.54 ± 0.67	0.033
C22:6n3	7.34 ± 1.77	8.11 ± 1.35	0.288
SFA	138.06 ± 30.72	110.06 ± 21.81	0.029
MUFA	58.57 ± 21.52	50.77 ± 16.58	0.376
PUFA	130.98 ± 32.18	97.42 ± 23.10	0.015
n6 PUFA	118.76 ± 29.45	84.33 ± 22.61	0.009
n3 PUFA	12.22 ± 3.77	13.10 ± 1.16	0.492
n6/n3	10.11 ± 2.20	6.43 ± 1.65	<0.001

UFA, sum of unsaturated fatty acids; MUFA, sum of monounsaturated fatty acids; PUFA, sum of polyunsaturated fatty acids; MX rabbit, Minxinan Black rabbit; LW rabbit, Laiwu Black rabbit.

**Table 2:** Key odor-active compounds exhibiting both significant breed-dependent differences and relative odor activity values (ROAV) ≥ 0.1 in longissimus dorsi muscle of Laiwu Black (LW) and Minxinan Black (MX) rabbits.

Compound	Odor_Threshold	OAV_LW	OAV_MX	ROAV_LW	ROAV_MX	*p*-Value
Hexanal	4	1.04	0.50	25.35	12.09	0.007
Ethanol	8	0.46	0.13	11.14	3.11	0.004
Hexanoic acid, ethyl ester	0.3	0.42	0.07	10.21	1.61	0.026
Pentanal	12	0.03	0.01	0.62	0.36	0.027
Thiophene, 2,5-dimethyl-	1	0.01	0.00	0.17	0.04	0.008
Octanoic acid, ethyl ester	5	0.00	0.00	0.10	0.02	0.040

## Data Availability

RNA-seq data have been submitted to the Genome Sequence Archive (GSA) (CRA027674) at the National Genomics Data Center and are publicly available at https://ngdc.cncb.ac.cn/gsa, accessed on 11 July 2025.

## Supplementary Materials

The following supporting information can be downloaded at https://www.mdpi.com/article/10.3390/ani16203162/s1; Table S1: Volatile organic compounds (VOCs) identified in the longissimus dorsi muscle of Laiwu Black (LW) and Minxinan Black (MX) rabbits and classified as potential characteristic aroma contributors; Table S2: Volatile organic compounds (VOCs) showing significantly different relative contents in the longissimus dorsi muscle between Laiwu Black (LW) and Minxinan Black (MX) rabbits; Table S3: Correlation network of differentially expressed genes (DEGs) and differentially abundant metabolites (DAMs) in the longissimus dorsi muscle of Laiwu Black (LW) and Minxinan Black (MX) rabbits; Table S4: Ten KEGG pathways co-enriched by differentially expressed genes (DEGs) and differentially abundant metabolites (DAMs) in the longissimus dorsi muscle of Laiwu Black (LW) and Minxinan Black (MX) rabbits; Table S5: Differential expression statistics (log_2_FC and FDR) of the PLD–PA–LPAR axis genes (*PLA2G7*, *PLD2*, *LPAR1*, *LPAR4*) between Laiwu Black (LW) and Minxinan Black (MX) rabbits; Table S6: Comparison of RNA-seq and RT-qPCR results for six key genes (*PLA2G7*, *PLD2*, *LPAR1*, *LPAR4*, *ELOVL5*, and *DGAT2*) in longissimus dorsi muscle of Laiwu Black (LW) and Minxinan Black (MX) rabbits (*n* = 10 per breed; mean ± SD; Welch *t*-test); Table S7: Primer sequences (forward and reverse) for the six target genes (*PLA2G7*, *PLD2*, *LPAR1*, *LPAR4*, *ELOVL5*, and *DGAT2*) and the reference gene GAPDH used for RT-qPCR validation; Figure S1: Quantitative outlier diagnostics for the metabolomics dataset (all 20 samples). Left: within-group Mahalanobis D^2^ in the top 5 PC space of the log2-transformed, pareto-scaled feature matrix (leave-one-out covariance estimation); dashed line, 99% χ^2^ (5 df) critical value (15.09). Middle: leave-one-out Hotelling’s T^2^ (95% critical value 263.3). Right: DModX from the PLS-DA model (critical value 21.45). The LW10 sample (red) is an extreme outlier by all three diagnostics, whereas no MX sample approaches the exclusion criterion; Figure S2: Permutation test of the O2PLS joint components (*N* = 200). For each iteration, sample pairing between the transcriptomic and metabolomic blocks was randomly permuted and the O2PLS model refitted; the test statistic S (sum of squared correlations between the two joint score vectors) is shown for the permuted models (blue histogram; null mean = 1.21) and for the observed model (red line, S = 1.77). The observed statistic exceeded all 200 permuted values (permutation *p* = 0.005); Figure S3: RT-qPCR validation of the six key differentially expressed genes (mean ± SD, *n* = 10). *, *p* < 0.05; **, *p* < 0.01.

## Abbreviations

The following abbreviations are used in this manuscript:

O2PLS	Two-way orthogonal partial least squares
PLD	Phospholipase D
LPAR	Lysophosphatidic acid receptor
PA	Phosphatidic acid
LPC	Lysophosphatidylcholine
ECM	Extracellular matrix
FASN	Fatty acid synthase
ELOVL2	ELOVL fatty acid elongase 2
ELOVL5	ELOVL fatty acid elongase 5
DAG	Diacylglycerol
TAG	Triacylglycerol
HMDB	Human Metabolome Database
