# Supplementary material for: Integrated Volatilomics, Metabolomics, and Transcriptomics Implicate PLD–PA–LPAR Signaling in Rabbit Meat Flavor Divergence
Source: Animals (Basel). 2026 Oct 9;16(20):3162. doi: 10.3390/ani16203162 (PMC13700755; doi:10.3390/ani16203162)
Supplement: Supplementary file 1 [file animals-16-03162-s001.zip › FigureS3.pdf]

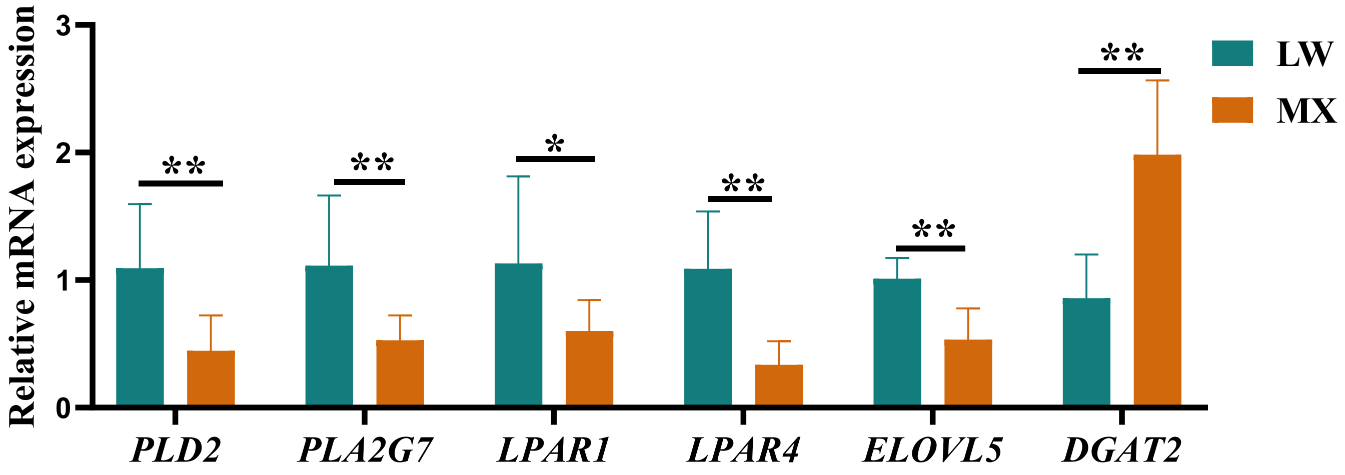

Relative mRNA expression
3
2
1
0
**
**
*
**
**
**
LW
MX
PLD2
PLA2G7
LPAR1
LPAR4
ELOVL5
DGAT2
